# Evaluation of a training program for life skills education and financial literacy to community health workers in India: a quasi-experimental study

**DOI:** 10.1186/s12913-020-06025-4

**Published:** 2021-01-08

**Authors:** Shantanu Sharma, Kanishtha Arora, Chandrashekhar, Rajesh Kumar Sinha, Faiyaz Akhtar, Sunil Mehra

**Affiliations:** 1 Department of Clinical Sciences, Lund University, Skåne University Hospital, S-20502 Malmö, Sweden & Assistant Director, MAMTA Health Institute for Mother and Child, Delhi, India; 2 Reproductive, Maternal, Newborn, Child and Adolescent Health Division, MAMTA Health Institute for Mother and Child, Delhi, India; 3 MAMTA Health Institute for Mother and Child, Delhi, India

**Keywords:** Community health workers, Communication, Decision making, Problem solving, Research, Training support, Time management

## Abstract

**Background:**

Accredited Social Health Activists (ASHA) are community health workers responsible for improving the health status of people by facilitating their access to healthcare services. The life skills of ASHA are known to be effective in negotiating behaviour change in the community; however, there has been a meagre focus towards improving them. Considering this gap, we adopted a comprehensive training program, known as Personal Advancement and Career Enhancement (P.A.C.E.), to empower ASHAs on life skills and financial literacy. The present study intends to assess the training program in two districts of Uttar Pradesh, India, by examining changes in knowledge, perceptions, and practices of ASHAs about life skills and financial literacy.

**Methods:**

We conducted a quasi-experimental, non-randomized, controlled study with pre-and post-test assessments. Data were collected on socio-demographic characteristics, knowledge, and practices related to life skills (communication skills, self-confidence, problem–solving and decision-making skills, time and stress management skills) and financial literacy. Additionally, change perceptions on gender-, life skills-, and savings-related practices at the personal, community, and workplace levels were assessed in the intervention group. Factor analysis was performed to obtain the change patterns by assessing the degree to which the four life skills, financial literacy, and change perceptions on practices were correlated. A general linear regression model was performed to assess associations among change pattern scores and socio-demographic variables.

**Results:**

We analyzed the data of 171 ASHAs (intervention group:86 and control group:85). There was a significant improvement in the average post-test scores of all the life skills and financial literacy in the intervention group (*p* < 0.001). Three distinct change patterns were found post-training in the intervention group. Factor 1 (high loadings for change perceptions on practices) was positively associated with ASHAs aged 38 and above and with experience of ≤12 years. On the contrary, the change in financial literacy and self-confidence scores was common among ASHAs with more than 12 years of experience.

**Conclusions:**

The P.A.C.E training program was found effective in improving the life skills and financial literacy of ASHAs in India.

**Supplementary Information:**

The online version contains supplementary material available at 10.1186/s12913-020-06025-4.

## Background

India, accounting for 17% of the world’s population, contributes to 19% of global maternal deaths and 21% of global childhood deaths [1]. However, it has made significant strides in improving maternal and child health coverage over the last decade, especially after the launch of the National Health Mission (NHM) program in 2005 [2]. The creation of a cadre of community health workers, Accredited Social Health Activists (ASHAs), across all villages, proved instrumental in improving maternal and child health care in India [3]. An ASHA is a woman resident in the community trained, deployed, and supported to function in her village to improve people’s health status by facilitating their access to healthcare services. Her job responsibilities are three-fold, i.e., the role of a link-worker (facilitating access to healthcare facilities and accompanying women and children), that of a community health worker (depot-holder for selected essential medicines and responsible for the treatment of minor ailments), and of a health activist (creating health awareness and mobilizing the community for a change). Close to 0.9 million ASHAs are currently trained and deployed across the country [4].

The national guidelines specify that ASHAs receive 23 days of training in the first year and 12 days of training in the subsequent years. The Ministry of Health & Family Welfare (MoHFW) has developed the training modules for ASHAs, focusing on healthcare-related aspects such as maternal, new born, and child health, reproductive health, gender-based violence, etc. [5]. However, content on improving their life skills like decision-making skills, problem-solving skills, time and stress management skills, and interpersonal communication skills is seldom addressed in the modules. Also, the health system lack mechanisms for continued learning and periodic upgrading of their skills. Due to the lack of such skills among ASHAs, they face difficulties in effectively negotiating behaviour change in the community [6]. Hence it is crucial to have a comprehensive capacity building program for ASHAs, which would increase their confidence and enable them to translate technical knowledge into practice. Studies have highlighted that increased employee self-efficacy positively impacts employee performance [6] and enhances competence and task-based self-esteem [7].

Given that there is a lack of the components of life skills education and financial literacy in the existing training modules of ASHA, we developed a training program. We adopted a structured, previously developed comprehensive learning program, known as Personal Advancement and Career Enhancement (P.A.C.E.) [8]. P.A.C.E. uses module-based learning to empower women on life skills.

Furthermore, limited studies have evaluated the effectiveness of ASHA’s training on life skills, and most of them are limited to communication skills [9, 10]. To the best of the author’s knowledge, no study has evaluated the effectiveness of ASHA’s training on other life skills, like decision-making, problem-solving, time and stress management, and financial literacy. In the backdrop of the aforesaid discussion, the present study intends to evaluate the training program by assessing the change in knowledge, perceptions, and practice of ASHAs about life skill education and financial literacy. We hypothesized that the training program would improve knowledge, perceptions, and practice of ASHAs, keeping assured the training fidelity.

## Methods

### Intervention

The P.A.C.E. training program’s goal was to equip ASHAs with life skills education and financial literacy for seeking improvement in their personal and professional outlook. There were four major modules in the P.A.C.E. program, apart from introductory and consolidation modules. The four major modules included were communication skills, problem-solving and decision-making skills, time and stress management skills, and financial literacy.

**Fig. 1:**
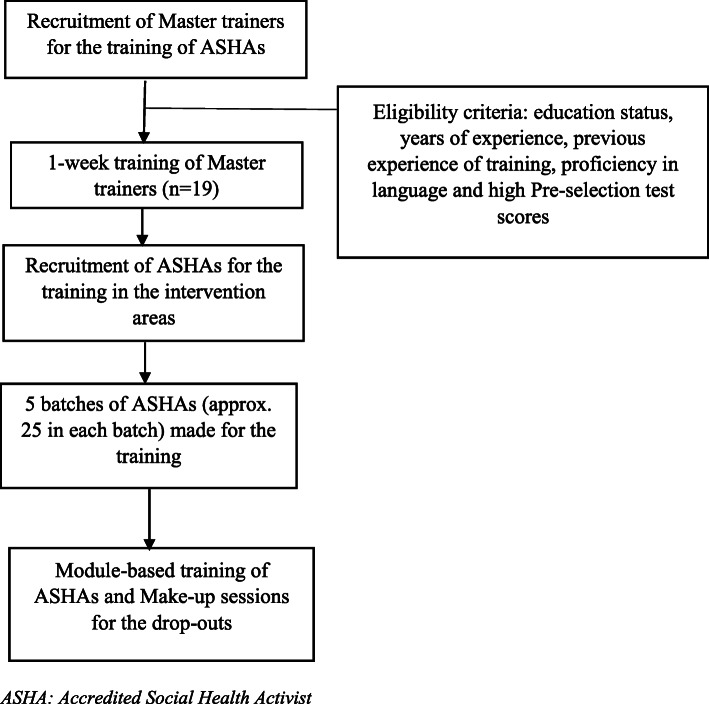
Flow chart of the key activities of the training program. This is the cascade model of training of ASHAs in the two intervention districts. The training was based on a 6 modules, including communication skills, problem-solving and decision-making skills, time and stress management skills, financial literacy, introduction and consolidation

The flow chart of the key activities in the training program is given in Fig. 1. We followed a cascade model of training (P.A.C.E. trainers created master trainers to train ASHA). We did pre-and post-test assessments for the master trainers (*n* = 19). Further, master trainers trained ASHAs on the six modules, and each training session lasted 6–7 h. A gap of 20 days between two sessions was kept purposively to let the contents of one be imbibed properly in the attendees. Hence, we covered all the modules in 6–7 months. All the trainings for ASHAs were done in the health facilities. The interactive modules had visuals, pictorials, games, and plays to understand the content better. The modules imparted 40 h of education, followed by enhanced technical training intended to help health workers to become more effective at work and improve their personal lives.

### Study design and sampling

We conducted a quasi-experimental, non-randomized, controlled study with pre-and post-test assessments among ASHAs of two selected districts. In each of the two selected districts, two control and intervention blocks were selected. The selection of intervention blocks was carried out randomly; simultaneously, control blocks were selected adjacent to the two intervention blocks in the respective districts. As this was a panel study, the same sampled respondents were provided training, and the same individuals were interviewed during the baseline and end line rounds. The individual outcomes’ trajectories in the intervention group could be assessed and compared with those of the control group due to the panel design. We adopted systematic random sampling. To have heterogeneity within the sample, the study delineated the sampling frame into a group, namely years of experience. The sample was distributed proportionately in each group for case and control arms, respectively. The training program was started in November 2018 in two districts of Uttar Pradesh in India, namely, Prayagraj and Varanasi. These two districts were selected because they had poor maternal and child health indicators [11]. The details of the training program are provided in supplementary file 1.

### Sample size and participation

$$ \mathrm{N}\frac{=2{\left({\mathrm{Z}}_{\alpha }+{\mathrm{Z}}_{\mathrm{b}}\right)}^2\times \mathrm{p}\left(1-\mathrm{p}\right)}{{\left({\mathrm{P}}_1-{\mathrm{P}}_0\right)}^2} $$

Where N = Sample size required for each group.

P_1_ = Probability of event in the intervention group = 75%.

P_0_ = Probability of event in the control group = 50%.

*p =* (P_1_ + P_0_)/2.

Z_α_ = Standard normal deviate corresponding to the level of significance (type I error rate).

Z_b_ = Standard normal deviate corresponding to the chance of not detecting the relative risk as significant (type II error rate).

A sample size of 60 per group was derived assuming that 50% of ASHAs had knowledge on life skills during the baseline and hypothesized that this would increase to 75% after the intervention at 95% confidence level and power of study as 80%. The sample size was equally divided in each intervention or control block. Within each block, we selected respondents randomly for the interview.

### Data collection

Ten trained investigators collected data. The baseline assessment was done in November 2018 and the end line in August 2019. We developed a structured questionnaire for the study to collect data in both survey rounds (supplementary file 2). The questionnaire had the following sections: a) socio-demographic characteristics; b) knowledge and practices related to life skills (communication skills, self-confidence, problem–solving and decision-making skills, time and stress management skills), and financial literacy; c) change perceptions on gender-, life skills-, and savings-related practices at personal, community, and workplace levels (only during the end line survey). The socio-demographic characteristics included age, years of schooling, years of experience working as ASHA, monthly income from the job, and social class ASHA belongs to.

### a. Life skills

The life skills were assessed through different scales. The communication skills scale consisted of 7 questions with scores ranging between 0 and 17. The self-confidence scale had six questions on a five-point Likert scale (‘no confidence’ to ‘very confident’) with scores ranging between 6 and 30. There were four questions in the problem-solving and decision-making scale, and the minimum and maximum scores were 0 and 11, respectively. Similarly, the time and stress management scale had five questions, and the scores ranged between 0 and 20. We summed up the individual questions’ scores in the life skills’ scales to calculate their aggregate scores. The reliability scores (Cronbach’s alpha score) of all the scales were found more than 0.7.

### b. Financial literacy

Assessment of financial literacy was based on five questions with minimum and maximum scores of the scale ranging between 0 and 24, respectively. The Cronbach’s alpha score of the scale was 0.76.

### c. Change perceptions

Additionally, during the post-test assessment, current practices and related perceptions of ASHAs from the intervention groups were also captured. Here, we specifically assessed general perceptions on P.A.C.E. training modules and change perceptions on gender-, life skills- (problem-solving, communication, and time management), and savings-related practices at the personal, community, and workplace levels. Separate scales for assessing change perceptions were developed. The questions in all the scales were based on a four-point Likert-scale. The responses varied from ‘completely agree’ to ‘completely disagree’. The questions’ responses on every scale were aggregated with ‘completely agree’ given a score of 2 and ‘agree’ a score of 1 and rest 0. The maximum and minimum scores of changes at the personal level varied between 0 and 22, community-level between 0 and 18, and the workplace between 0 and 14. The reliability scores (Cronbach’s alpha score) of all the scales were observed more than 0.85. For assessing general perceptions on P.A.C.E. training modules, the participants were asked to rate the quality of the training on a scale from 1 to 10.

The questionnaires were standardized, translated into the local language (Hindi), and field-tested before data collection. We used Computer Assisted Personal Interview (CAPI) for collecting quality real-time data during both survey rounds. To collect quality data, two supervisors, one in each district, were assigned to randomly back-check and spot-check 10% of all interviews during both rounds.

### Data analysis

The data were analyzed using the IBM SPSS Statistics for Windows version 24.0 (IBM Corp., Armonk, N.Y., USA). Descriptive data were expressed as frequency or percentages for categorical variables and mean (Standard Deviation, SD) or median (Interquartile Range, IQR) for continuous variables. The paired t-test (or Wilcoxon signed-rank test for medians) was conducted to assess the differences between average pre-and post-test scores in the intervention and control groups of life skills and financial literacy. Given that there were eight outcomes in which we sought the change, namely, four life skills, financial literacy, and change perceptions at three levels, we considered factor analysis. The factor analysis helped us derive different change patterns in the participants due to training. We used this method to assess the degree to which life skills, financial literacy, and change perceptions in the intervention group were correlated and derive a new set of composite variables. These new set of composite variables, not related to each other, represent discrete change patterns due to training. Only the change patterns with eigenvalues > 1.0 were included in the analysis. The variables that loaded highly (| > 0.30|) in varimax rotated change patterns were shown in the analysis. The Kaiser-Meyer-Olkin (KMO) measure reached the acceptable limit of 0.6, and Bartlett’s test of sphericity was significant (*p* < 0.001), meaning thereby that the data were suitable for factor analysis.

We performed a general linear regression model to assess the association between the change pattern scores and socio-demographic variables using main-effect analysis. Standard regression coefficients (β) and 95% confidence intervals were used to depict the strength and precision of associations. A two-sided *p*-value < 0.05 was considered statistically significant.

### Ethical considerations

MAMTA Ethical Review Board granted ethical approval for the study. We obtained written informed consent from all the study participants.

## Results

### Socio-demographic profile

**Fig. 2:**
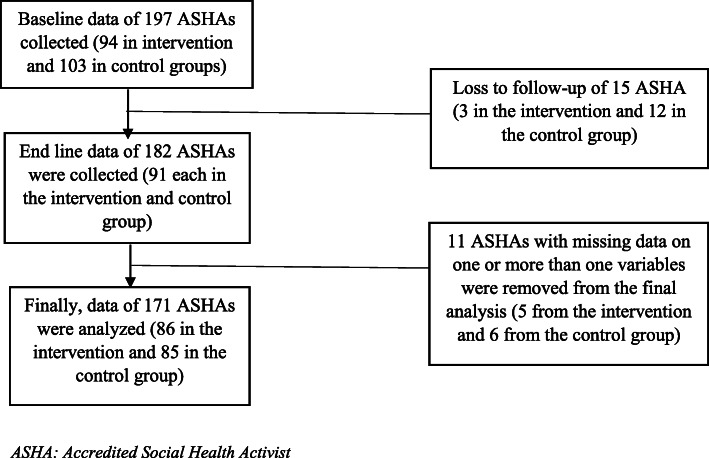
Flow chart of the study participants enrolled and analyzed

Initially, baseline data of 197 ASHAs were obtained, constituting 94 from the intervention arm and 103 from the control arm (Fig. 2). However, due to the loss to follow-up and missing data of some of the ASHAs, we could analyse 171 ASHAs (intervention arm: 86 and control arm: 85). Table 1 describes the socio-demographic profile of the ASHAs that participated in the study. The majority of the ASHAs in the intervention (64%) group aged ≤38 (Table 1). Nearly two-thirds of the ASHAs (64%) in the intervention group and a half in the control group received more than 11 years of schooling. Around 68% of ASHAs, in total, had ≤12 years of experience. Nearly all the ASHAs in both intervention and control groups belonged to scheduled caste or tribe or other backward classes.

**Table 1:** Socio-demographic characteristics of the study participants in the two groups

Characteristics	Intervention group ***n*** = 86 n(%)	Control group ***n*** = 85 n(%)	Total ***n*** = 171 n(%)
**Age groups**			
≤ 38 years	55 (64.0)	37 (43.5)	92 (53.8)
> 38 years	31 (36.0)	48 (56.5)	79 (46.2)
**Years of schooling**			
≤ 11 years	31 (36.0)	42 (49.4)	73 (42.7)
> 11 years	55 (64.0)	43 (50.6)	98 (57.3)
**Years of experience**			
≤ 12 years	56 (65.1)	60 (70.6)	116 (67.8)
> 12 years	30 (34.9)	25 (29.4)	55 (32.2)
**Monthly income (Indian rupees, INR)**			
≤ 2500 INR	47 (54.7)	49 (57.6)	96 (56.1)
> 2500 INR	39 (45.3)	36 (42.4)	75 (43.9)
**Social class**			
SC/ST	46 (53.5)	45 (53.0)	91 (53.2)
OBC	39 (45.3)	34 (40.0)	73 (42.7)
General	1 (1.2)	6 (7.0)	7 (4.1)

*Abbreviations*: *OBC* Other Backward class, *SC* Scheduled caste, *ST* Scheduled tribe

### Pre-and post-test scores of four life skills and financial literacy

There was a significant increase in the average post-test scores of communication (*p* < 0.001), self-confidence (*p <* 0.001), problem-solving and decision-making (*p <* 0.001), and time and stress management (*p <* 0.001) skills in the intervention group (Table 2). Although we noted a significant difference in the average post-test scores of problem-solving and decision-making skills in the control group (*p* = 0.02), the scores had actually decreased. The mean scores of financial literacy increased post-intervention in both groups; however, the increase was higher in the intervention (*p* < 0.001) than in the control group (*p <* 0.001).

**Table 2:** Distribution of the scores of four life skills and financial literacy among ASHAs before and after the intervention in the intervention and control groups

Characteristics	Intervention group (***n =*** 86)			Control group (***n =*** 85)		
	Pre-test	Post-test	***P*** value	Pre-test	Post-test	***P*** value
Communication skills mean (SD)	6.1 (1.7)	11.5 (2.1)	**< 0.001**	6.1 (2.1)	6.2 (2.0)	0.8
Confidence skills Mean (SD)	15.9 (4.0)	24.0 (3.7)	**< 0.001**	18.5 (5.9)	19.5 (4.4)	0.2
Problem-solving and decision-making skills^a^ Median (IQR)	2.0 (0–4.0)	8.0 (7.0–9.0)	**< 0.001**	2.0 (0–5.0)	0 (0–3.0)	**0.02**
Time and stress management skills^a^ Median (IQR)	4.5 (2.0–6.0)	10.0 (8.0–13.0)	**< 0.001**	5.0 (1.0–7.0)	5.0 (2.0–7.0)	0.3
Financial literacy	4.6 (2.4)	16.2 (3.3)	**< 0.001**	6.0 (3.2)	9.9 (4.2)	**< 0.001**

*Abbreviations*: *SD* Standard Deviation, *IQR* Interquartile Range

^a^*Wilcoxon signed-rank test was run*

*P value < 0.05 was considered significant and highlighted in bold*

### ASHA’s change perceptions on gender-, life skills- and savings-related practices at the personal, community, and workplace levels

Around 93% (80 out of 86) of participants in the intervention group attended the session on communication skills, 92% (79 out of 86) problem-solving and decision-making, and 89.5% (77 out of 86) time and stress management. Seventy out of 86 (81.4%) ASHAs attended the session on financial literacy. Approximately 80% of ASHAs (69 out of 86) rated the quality of the sessions ≥7 on a scale of 1–10. Furthermore, 80 out of 86 (93.0%) ASHAs would like to participate again in training and recommend this training to others. The median (IQR) score of the change perceptions on gender-, life skills-, and savings-related practices at the personal level was 12 (6–15). Similarly, the median (IQR) scores of the change perceptions at community and workplace levels were 11 (5–14) and 9 (5–11), respectively.

### Factor analysis of change patterns due to training

The first three components that explained the largest proportions of variance in the change patterns due to training had eigenvalues more than 1.0. These components were retained as three discrete change patterns due to training among ASHAs (Table 3). Together, these three patterns explained 79.8% (43.9, 18.8, and 17.0%, respectively) of the variation in change patterns. Factor 1 was characterized by high factor loadings for change perceptions on gender-, life skills-, and savings-related practices at the personal, community, and workplace levels. Hence, we named factor 1 as ‘enhanced change perceptions.’ Factor 2 was characterized by high loadings for three life skills (communication skills, problem–solving and decision-making skills, time and stress management skills). Factor 2 was labelled as ‘enhanced change in life skills.’ Factor 3 was characterized by high loadings for self-confidence and financial literacy. Hence, it was labelled as ‘enhanced change in confidence and financial literacy.’

**Table 3:** Factor loadings for the four life skills, financial literacy and change perceptions at personal, community, and workplace levels that loaded highly (| > 0.30|) in varimax rotated components

Components	Factor 1	Factor 2	Factor 3
Eigen value	3.51	1.51	1.36
% variance explained	43.91	18.88	17.03
Change perceptions at personal level	0.94	–	–
Change perceptions at community level	0.94	–	–
Change perceptions at workplace	0.91	–	–
Communication skills	–	0.72	–
Problem-solving and decision-making skills	–	0.85	–
Time and stress management skills	–	0.79	–
Self-confidence skills	–	–	0.89
Financial literacy	–	–	0.82

### Regression analysis

As shown in Table 4, the ‘enhanced change perceptions’ factor was inversely associated with the younger age group (≤38 years) of ASHAs (*p* = 0.004). An experience of ≤12 years among ASHAs was positively associated with the ‘enhanced change perceptions’ factor (*p*-value =0.005), and the ‘enhanced change in life skills’ factor (*p-*value = 0.01), and inversely associated with the ‘enhanced change in confidence and financial literacy’ factor (*p =* 0.004).

**Table 4:** General linear model for the change patterns’ associations with sociodemographic variables

Socio-demographic variables	Factor 1	Factor 2	Factor 3
	β (95% CI)	β (95% CI)	β (95% CI)
Age			
≤ 38 years	**−0.642 (−1.077, −0.207)**	0.430 (−0.005, 0.866)	−0.121 (−0.552, 0.311)
> 38 years	*Reference*	*Reference*	*Reference*
Years of schooling			
≤ 11 years	0.104 (−0.328, 0.536)	0.403 (−0.029, 0.836)	− 0.229 (− 0.651, 0.200)
> 11 years	*Reference*	*Reference*	*Reference*
Years of experience			
≤ 12 years	**0.660 (0.203, 1.116)**	**0.672 (0.215, 1.129)**	**−0.677 (−1.129, − 0.224)**
> 12 years	*Reference*	*Reference*	*Reference*
Monthly salary			
≤ 2500 INR	−0.055 (− 0.491, 0.381)	−0.422 (− 0.859, 0.014)	−0.323 (− 0.755, 0.109)
> 2500 INR	*Reference*	*Reference*	*Reference*

*β (95% CI): Standard regression coefficients (β) and 95% confidence intervals*

*R*^*2*^ *for factor 1 regression analysis = 13.7%, R*^*2*^ *for factor 2 regression analysis = 13.4%; R*^*2*^ *for factor 3 regression analysis = 15.1%*

*P-value was < 0.01 for the associations highlighted in bold*

*Factor 1: Enhanced Change Perceptions; Factor 2: Change in Life Skills; Factor 3: Enhanced change in Self-Confidence and financial literacy*

## Discussion

To the best of our knowledge, this is one of the first studies that evaluated the life skills training and financial literacy of ASHAs (community health workers) in India. There was a statistically significant improvement in the participants’ post-test scores in the intervention group in all life skills (communication skill, self-confidence, problem-solving and decision-making skill, time and stress management skill) and financial literacy. Three distinct change patterns were found post-training in the intervention group. The enhanced change perceptions were positively associated with health workers aged > 38 and with experience of ≤12 years. On the contrary, the change in financial literacy and self-confidence scores was common among health workers with > 12 years of experience.

As stated in the national guidelines and previous literature, ASHAs should be between 25 and 45 years of age and have completed 8 years of schooling at the time of selection [12, 13]. The participants from the intervention group in our study had similar age and education distribution. A higher presentation of ASHAs from the scheduled caste or tribes reflects the system’s response to enhanced coverage and quality delivery of services to poor and marginalized people by skilled workers from their community [14]. Furthermore, one-third of the participants in the present study had more than 12 years of experience. The increasing years of experience bring to ASHAs an increased social capital and networking within the health system and increased skills and performance in delivering outcomes [15]. Started in 2005, the ASHA program is still evolving in India and has been viewed from different lenses. The empowering views of the program are improved respect and dignity to health workers in the community, and the disempowering lens finds a lack of support and political contradictions from the health systems [15].

Life skills, essential for professional practice, help health workers deal with patients face-to-face, make correct decisions in difficult situations, and improve their performance and career prospects [16]. Community health workers, acting as agents of social change, need to feel empowered and equipped with life skills such as communication, problem-solving, etc. [17]. However, the life skills gap in community health workers has been poised as a critical challenge that impacts their performance and lack of motivation, self-worth, job satisfaction, and a high attrition rate [16, 18, 19]. Building on the need to address this gap, the present study provided evidence of the effectiveness of a structured training program in improving the life skills and practices of ASHAs in the community. We observed a significant improvement in all the life skills and financial literacy post-training. P.A.C.E training program conducted for another set of professionals (non-health workers) in India and neighbouring countries reported similar results with improved gender issues/relations, productivity, confidence, and better time management and communication skills [20]. Other studies have evaluated the life skills training program for community health workers with different training structures or strategies and found considerable improvement in their performance, decision-making capacity, patients dealing, time management, self-confidence, and interpersonal skills [16, 19].

P.A.C.E. training program gains an advantage over other programs by being holistic- focusing on women’s work and personal advancement and sustainable- integrating the program into existing internal operations [20]. Moreover, the program is designed to be flexible, adaptable, and contextualized for the setting in which it is implemented.

Financial literacy is the ability to make informed judgments and take effective decisions regarding the use and management of money. It can help health workers avoid financial distress and achieve financial security with improved physical and mental well-being [21]. However, there is a huge gap in the financial literacy levels among health workers [22]. Previous studies highlighted that financial literacy is poor, even among educated and working women [23, 24]. We found a significant increase in the financial literacy scores in the intervention group from the baseline. However, some increase was noted in the control group as well. The plausible explanation for such an increase in the control group could be an exposure to another training program [25]. However, further inquiry is required to better explain the increase in the level of financial literacy in the control group.

Female health workers experience unequal gender relations with the community and other health cadres. They need to exercise agency to deftly balance the demands and supply of essential services in the community [15]. The training on gender dynamics and relations helps community health workers to navigate and negotiate the difficulties and dilemmas at all levels, personal, community, and workplace. Our findings accord with this thought, and ASHAs realised that their understanding of gender improved after the training program.

Finally, in highlighting the socio-demographic attributes of the change patterns observed, our findings demonstrate that ASHAs elder in age but with experience ≤12 years have increased probability of acquiring life skills and change perceptions at personal, community, and workplace levels. This clearly affirmed the previous studies’ observations that the health workers’ performance is often related to increasing age and experiences regarding relations and power [15, 26, 27]. A greater probability of increased financial literacy and self-confidence scores among ASHAs with more than 12 years of experience may reflect the need and outcome of savings in the last 10–12 years.

Contrary to the published literature, we could not find the effect of increasing years of education on any of the three change patterns [26]. However, we concur with the findings from a review, which demonstrated that health workers with low levels of formal education could be trained effectively for acquiring skills and knowledge [28]. We could not find statistically significant improvement in financial literacy or life skills among ASHAs with higher incomes. David McCoy and his colleagues’ work on adequacy of incomes for health workers demonstrates that low pay can cause decreased retention, increased dissatisfaction, and loss of motivation among health workers [29]. The ASHAs in the study did not receive a salary and depended on performance-linked incentives [15]. We argue that regular monthly income and secured salary would improve performance and job satisfaction [30].

Similar studies globally have identified the importance of communication skills to improve the patient-provider relationship and enhance service delivery. Moreover, such trainings have been found to improve patient outcomes [31]. There is a highlighted need for building the life skills of community health workers besides improving health literacy and technical information [32]. Improved self-confidence emanating from increased knowledge and support will further ASHAs interpersonal relationships and enhanced trust and social standing in the community [10].

### Limitations of the study

The results of the study should be interpreted given certain limitations. Firstly, the assessment of the immediate effects of the training (knowledge, practices, and perceptions) was done without assessing its effect on the end-users to whom ASHAs serve in the community. Secondly, the study was conducted on a small scale in localized settings that may limit the generalization of findings. Lastly, due to budgetary and time constraints, more specific models of life skill training evaluation were not undertaken, such as Kirkpatrick’s model and People Styles model [33].

## Conclusions

The P.A.C.E training program was found effective in improving the life skills and financial literacy of community health workers (ASHAs) in India in the intervention group. The module-based training spread over 6 months improved not only the knowledge but also the perceptions and practices related to gender-, life skills-, and savings- at the personal, community, and workplace levels. Age and years of experience of workers influenced their improved knowledge and perceptions related to life skills and financial literacy. We propose to organize such trainings for health workers at the beginning of their career for greater effectiveness and sustainability of learnings.

## Supplementary Information

**Additional file 1: Supplementary file 1.** Key issues covered under 4 core training modules of P.A.C.E, namely communication skills, problems-solving and decision-making skills, time management skills, and financial literacy, master trainer’s profile, and distribution of time for the different modules of the training.

**Additional file 2: Supplementary file 2.** Questionnaire. The questionnaire employed in the interviews with community health workers (ASHA) for pre- and post-test assessments

## Data Availability

The data can be made accessible to the readers on request to the corresponding author**.**

## Abbreviations

ASHA: Accredited Social Health Activist
CAPI: Computer Assisted Personal Interview
IQR: Interquartile Range
MoHFW: Ministry of Health and Family Welfare
NHM: National health Mission
P.A.C.E.: Personal Advancement and Career Enhancement
SD: Standard Deviation
