# Supplementary material for: Evaluation of a training program for life skills education and financial literacy to community health workers in India: a quasi-experimental study
Source: BMC Health Serv Res. 2021 Jan 8;21:46. doi: 10.1186/s12913-020-06025-4 (PMC7796593; doi:10.1186/s12913-020-06025-4)
Supplement: Supplementary file 1 — Additional file 1: Supplementary file 1. Key issues covered under 4 core training modules of P.A.C.E, namely communication skills, problems-solving and decision-making skills, time management skills, and financial literacy, master trainer’s profile, and distribution of time for the different modules of the training. [file 12913_2020_6025_MOESM1_ESM.docx]

P.A.C.E. training program

**CORE-Training Modules**

1. Communication skills: Key issues covered include Basics, process and dynamics of communication, and communication at home, community, and workplace
2. Problems-solving and decision-making skills: Key issues covered include basics, consensus building, application of problem-solving and decision-making.
3. Time management skills: Key issues covered include managing time, refining goals, stress management, and positive thinking.
4. Financial literacy: Key issues covered include importance of savings, saving options, and financial planning.

**Master trainer’s profile**

There were 5 male trainers and rest female. The mean years of experience of the trainers was 7 years. All were proficient in the local language and 8 were proficient in English as well.

**Pre- and Post-test assessment of master trainers**

There were 8 sections, including communication skills, Problem-solving and decision-making skills, time and stress management skills, water, sanitation and hygiene, gender and reproductive health, financial literacy, legal literacy, and execution excellence. There were 10 questions in each of these sections.

**Distribution of time for the different modules of the training**

| **Weeks** | **Hours** | **Modules** | |
| --- | --- | --- | --- |
| Week 1 | 4.5 | Introduction | Communication skills Part-1 |
| Week 2 | 7.5 | Communication skills Part-2 | |
| Week 3 | 6 | Problem-solving and decision-making Part-1 | |
| Week 4 | 7.5 | Problem-solving and decision making Part-2 | Time and Stress management Part-1 |
| Week 5 | 7.5 | Time and Stress management Part-2 | |
| Week 6 | 7.5 | Financial literacy | Concluding session |
|  | **40.5** |  |  |
