# Supplementary material for: Evaluation of a training program for life skills education and financial literacy to community health workers in India: a quasi-experimental study
Source: BMC Health Serv Res. 2021 Jan 8;21:46. doi: 10.1186/s12913-020-06025-4 (PMC7796593; doi:10.1186/s12913-020-06025-4)
Supplement: Supplementary file 2 — Additional file 2: Supplementary file 2. Questionnaire. The questionnaire employed in the interviews with community health workers (ASHA) for pre- and post-test assessments [file 12913_2020_6025_MOESM2_ESM.docx]

**Baseline Study for P.A.C.E Gap Inc.**

**_____________________________________________________**

**Interviewer Administered Questionnaire for ASHA**

**Purpose of the study**

Namaste! My name is ________________, and I am working as investigator for a study on adolescent health and development by MAMTA Health Institute for Mother and Child. MAMTA Health Institute for Mother and Child is a non-government, not for profit organization in India, with its office in Delhi and Lucknow. This study is being conducted in two districts of Uttar Pradesh.

We are conducting this study to evaluate the effect of implementing Gap Inc. P.A.C.E. program in terms of knowledge, perception and practice among ASHA and adolescent girls (14 – 17 years) in personal and professional life skills.

**Procedures**

We have selected your area for the study. We would like to do a survey with you. For this, we will ask a series of questions developed to understand the knowledge, perception and practice among ASHA. If you agree, then you will be one among many ASHAs to participate in the interview. The complete interview will require about 20 minutes.

**Confidentiality**

We will do everything to protect the confidentiality of what you share during the interview. Whatever is said will not be shared with anyone outside the research team or discussion group and no name will be taken to help preserve the confidentiality. No name or any identification will be mentioned in report or any other document.

**Risks and Precautions**

The interview has no risk as such. However, during the interview, you may feel uncomfortable or upset while hearing about or discussing some of the topics in the study. If so, you do not have to answer the questions. You can withdraw from the interview at any point. Your refusal to participate will not affect you adversely.

If you feel upset or uncomfortable, you can talk to me or District/State Coordinator (contact details given in the list provided). If you want, you can call us to clarify any issues. We have also provided you a sheet with the contact information of the research and project team members who can assist you to discuss these issues.

**Benefits**

You will not be paid for your participation in the survey however we believe that you will enjoy and benefit from discussing topics that are important and relevant to life skills. The information you provide will also help strengthen the program

**Right to Refuse or Withdraw**

Participation in this research is voluntary, and you are free to decide to participate. If you decide to participate, you can still choose not to answer any question or join in the discussion of any particular topic, or even decide to go out of the study/program at any time. If you choose to do this, nothing will happen to you or your family and you will continue in your job and receiving services from Government schemes.

If you have any question about the study and would like a face-to-face meeting with a member of the research team, please call (*name and contact number of District/ State coordinator).* You can also ask questions over the phone at any point.

Before you decide whether to give your consent, it is important that you feel like you have received a clear and detailed explanation about your participation in this study. You have the right to ask, and have answered, any questions you may have about this research. If you have questions before or after signing the form, you may contact District/ State Coordinator. You can have a copy of the consent form, if you wish.

Do you agree to participate?

Yes / No

**B. Certificate of Consent**

I have read or have been read the above considerations regarding my participation in the study. I have been given a chance to ask questions and the questions have been answered to my satisfaction.

I understand that my records will be kept private and that I can stop the survey/discussion at any time. I also understand that my decision to stop the discussion will not affect me adversely.

I agree to this survey/discussion.

Signature of participant ____________________________

If oral consent, Signature of Witness ___________________

Date ________________

**Investigator’s statement**

I, the undersigned, have explained to the participant the procedures to be followed in the study and the risks and benefits involved. I have also provided my mobile number so that he/she may contact me at any time if required.

Name of the discussant _____________________________

Signature of the discussant _______________________ Date _________________

| **MAMTA Health Institute for Mother and Child, New Delhi** | | | |
| --- | --- | --- | --- |
| **Identification** | | | |
| 1. | State | Uttar Pradesh | 1 |
| 2. | District | Allahabad  Varanasi | 1  2 |
| 3. | Name of the Block | …………………….. |  |
| 4. | Serial number of the respondent | ______ ______ |  |

**Section 1: SOCIO – DEMOGRAPHIC INFORMATION**

*In this section, we want to ask you some questions about your schooling, employment and marital status.*

| **Q. No** |  | **Category** | **Code** | **Skip** |
| --- | --- | --- | --- | --- |
| 101. | Name of the Respondent |  |  |  |
| 102. | Current age of the respondent | Age in completed year |  |  |
| 103. | What is the highest standard you completed? | (in years)  __ |  |  |
| 104. | Religion | Hindu  Muslim  Sikh  Christian  Others specify | A  B  C  D  O |  |
| 105. | Caste | SC  ST  OBC  General Caste  Others | A  B  C  D  E  O |  |
| 106. | How many years you have experienced in this profession? | ……………….. |  |  |
| 107. | What is your average monthly honorarium/ salary? Incentives | ……………….. |  |  |
| 108 | Did you take part in the P.A.C.E. training program? | Yes  No | A  B | If B then, don not ask Section 6 |

**Section 2: COMMUNICATION**

*In this section, we want to ask you some questions about your communication skills and practice.*

| **Q. No.** |  | **Category** | **Code** | **Skip** |
| --- | --- | --- | --- | --- |
| 201. | Do you feel confident to bring about a desired change among the community members whom you communicate with? | Yes  No  Can’t say | A  B  C |  |
| 202. | Are you able to negotiate for an issue that is of high importance to you with your seniors at work place? | Yes  No  Can’t say | A  B  C |  |
| 203. | Are you able to put across clearly about what you like and dislike to the family members? | Yes  No  Can’t say | A  B  C |  |
| 204. | According to you what are the components of effective communication?  **MULTIPLE RESPONSE**  Do not prompt replies | Engaged Listening  Stress management  Appropriate non-verbal body language  Communicate with self-confidence  Others specify  Don’t know | A  B  C  D  O  N |  |
| 205. | Can you list out some of the problems faced during effective communication?  **MULTIPLE RESPONSE**  Do not prompt replies | Use of unfamiliar / complicated words  Emotional and social norms  Lack of attention / interest  Language differences  Physical disability  Appropriate time  Others specify  Don’t know | A  B  C  D  E  F  O  N |  |
| 206. | Gender discrimination is a challenge for effective communication? | Yes  No  Can’t say | A  B  C |  |
| 207. | What are different styles of communication?  **MULTIPLE RESPONSE** | Aggressive  Submissive  Assertive  Others specify  Don’t know | A  B  C  O  N |  |
| 208. Now, I would like to ask some questions on the basis of your confidence, please rate your confidence level | | | | |
|  | **Questions** | | **Rate your confidence from 1 to 5**  **1= NO confidence 5= VERY confident** | |
| a. | How confident are you about your ability to talk with people? | |  | |
| b. | How confident you feel when people include you in conversations? | |  | |
| c. | How much confident are you about your ability to speak on the telephone? | |  | |
| d. | How confident you feel that people understand you when you talk? | |  | |
| e. | How much comfortable you feel yourself while talking to your supervisor/ASHA sangini at work? | |  | |
| f. | How comfortably you speak to the person with other gender? | |  | |

**Section 3: PROBLEM SOLVING AND DECISION MAKING**

*In this section, we want to ask you some questions about your problem solving and Decision-making skills.*

| **Q. No** |  | **Category** | **Code** | **Skip** |
| --- | --- | --- | --- | --- |
| 301. | Do you know steps of problem solving | Yes  No  Can’t say | A  B  C |  |
| 302. | If yes, what are those steps  **MULTIPLE RESPONSE** | Avoid ‘First Emotion  Define The Problem & Identify Root Cause  Consider ALL Possible Options  Select the BEST Option  Implement & Review for improvements  Others specify | A  B  C  D  E  O |  |
| 303. | Do you feel that coping strategies should be applied for problem solving? | Yes  No  Can’t say | A  B  C |  |
| 304. | If yes, what are the coping strategies that you are practicing yourself to reduce stress?  **MULTIPLE RESPONSE** | Avoidance  Over-reliance on others  Balanced approach  Over-reliance on self  Others specify | A  B  C  D  O |  |

**Section 4: TIME AND STRESS MANAGEMENT**

*In this section, we want to ask you some questions about your time and stress management.*

| **Q.no** |  | **Category** | **Code** | **Skip** |
| --- | --- | --- | --- | --- |
| 401. | Are you able to prioritize multiple things to be done as part of your work? | Yes  No  Can’t say | A  B  C |  |
| 402. | If yes, how do you accomplish or get things done?  **MULTIPLE RESPONSE** | Maintaining a daily “to do” task list  Prioritization  Negotiation wherever required  Saying no to certain “not so important” things  Multitasking  Delegation  Seeking support  Breaking down bigger jobs into doable tasks  Others specify | A  B  C  D  E  F  G  H  O |  |
| 403. | Do you feel that coping strategies should be followed to reduce stress? | Yes  No  Can’t say | A  B  C |  |
| 404. | If yes, what are the coping strategies that you are practicing yourself to reduce stress?  **MULTIPLE RESPONSE** | Positive thinking  Talk/ Go to a friend  Take nutritional food  Share your problem with others  Sleep  Do relaxation exercises  Take part in sports or leisure activities you like  Time management  Accepting things which you can’t control  Others specify | A  B  C  D  E  F  G  H  I  O |  |
| 405. | To what extent, do you overcome the stress by utilizing stress management  facilities and practicing coping strategies? | To a great extent  To some extent  No change | A  B  C |  |

**Section 5: FINANCIAL LIYERACY**

*In this section, we want to ask you some questions about knowledge and practice on Financial Literacy.*

| **Q. No** |  | **Category** | **Code** | **Skip** |
| --- | --- | --- | --- | --- |
| 501. | What are the benefits of savings?  **MULTIPLE RESPONSE** | Provides financial security  Increases self-confidence  Creates better relationships, peace, and harmony  Fulfills basic needs  Improves quality of life  Equips one to deal with emergencies and crises  Teaches one to spend wisely—less than the earning  Others specify  Don’t know | A  B  C  D  E  F  G  O  N |  |
| 502. | What are the risks of not saving?  **MULTIPLE RESPONSE** | Leads to debt  Creates stress and tension  Disturbs peace and harmony  Decreases self-confidence  Leaves one incapable of coping with emergencies and unexpected expenses  Others specify  Don’t know | A  B  C  D  E  O  N |  |
| 503. | What are the different options available for savings?  **MULTIPLE RESPONSE** | Banks  Post office  Self-help groups  Insurance  Chit funds  Mutual funds  Buying gold, silver, land, and property  Others specify  Don’t know | A  B  C  D  E  F  G  O  N |  |
| 504. | What are saving options are available in a bank  **MULTIPLE RESPONSE** | Savings Account  Recurring Deposit  Term Deposit/Fixed Deposit  Others specify  Don’t know | A  B  C  O  N |  |
| 505. | What are financial planning goals?  **MULTIPLE RESPONSE** | Goal setting and family arrangements  Budgeting  Others  Don’t know | A  B  C  N |  |

**Section 6: SESSION ATTENDED AND PRACTICES**

| 601 | Which module did you participate in?  **(Multiple response possible)** | Communication  Problem solving and decision making  Time and stress management  Financial literacy | | | | A  B  C  D |  |
| --- | --- | --- | --- | --- | --- | --- | --- |
| 602 | Which module did you like the most?  **(Multiple response possible)** | Communication  Problem solving and decision making  Time and stress management  Financial literacy | | | | A  B  C  D |  |
| Now, I shall ask you some questions related to your behavior and perception about the training and P.A.C.E. program. I shall read some statements; you are requested to give your response based on your agreement and disagreement for each of the statement, starting from completely agree to completely disagree. | | | | | | | |
| **Sr. No.** | **Statements** | | **Completely agree** | **Agree** | **Disagree** | | **Completely disagree** |
| **603 (i)** | I often think to use learnings of P.A.C.E. training program in my day to day life. | | 1 | 2 | 3 | | 4 |
| **603 (ii)** | I feel more aware about the matters relating to gender bias, stereotype and discrimination in my family. | | 1 | 2 | 3 | | 4 |
| **603 (iii)** | I observe the gender bias phenomenon which I earlier use to overlook. | | 1 | 2 | 3 | | 4 |
| **603 (iv)** | I freely discuss on gender and reproductive issues and other problems with family and friends. | | 1 | 2 | 3 | | 4 |
| **603 (v)** | Now I use gender sensitive language to communicate with my family members.. | | 1 | 2 | 3 | | 4 |
| **603 (vi)** | I have become more responsible in my attitude towards male as well as females. | | 1 | 2 | 3 | | 4 |
| **603 (vii)** | I feel more confident than before in talking about gender discrimination in the family. | | 1 | 2 | 3 | | 4 |
| **603 (viii)** | I have started raising my voice against gender discrimination at home. | | 1 | 2 | 3 | | 4 |
| **603 (ix)** | I talk to both the gender with ease. | | 1 | 2 | 3 | | 4 |
| **603 (x)** | I prepare my own curriculum vitae | | 1 | 2 | 3 | | 4 |
| **603 (xi)** | I have started saving money for the future | | 1 | 2 | 3 | | 4 |
| I shall ask you some questions about your behavior and perception in the community about the training and P.A.C.E. program. Again, I shall read some statements; you have to give your response based on your agreement and disagreement for each of the statement, starting from completely agree to completely disagree. | | | | | | | |
| **Sr. No.** | **Statements** | | **Completely agree** | **Agree** | **Disagree** | | **Completely disagree** |
| **604 (i)** | I am using learnings of P.A.C.E. module during discussions in my community. | | 1 | 2 | 3 | | 4 |
| **604 (ii)** | I feel more aware about the matters relating to gender bias, stereotype and discrimination in my community. | | 1 | 2 | 3 | | 4 |
| **604 (iii)** | I freely discuss on gender and reproductive issues and other problems with community members. | | 1 | 2 | 3 | | 4 |
| **604 (iv)** | I feel more confident than before in talking about gender discrimination in my community. | | 1 | 2 | 3 | | 4 |
| **604 (v)** | I have started sensitizing community regarding issues related to gender based discrimination. | | 1 | 2 | 3 | | 4 |
| **604 (vi)** | I talk to people in the community regarding savings and its future needs | | 1 | 2 | 3 | | 4 |
| **604 (vii)** | I try to manage my issues with ease in the community | | 1 | 2 | 3 | | 4 |
| **604 (viii)** | Now I try to manage with my problems by own. | | 1 | 2 | 3 | | 4 |
| **604 (ix)** | After the training, I realize that I started managing my time well. | | 1 | 2 | 3 | | 4 |
| Finally, I shall ask you some questions about your behavior and perception in the workplace about the training and P.A.C.E. program. Again, I shall read some statements; you have to give your response based on your agreement and disagreement for each of the statement, starting from completely agree to completely disagree. | | | | | | | |
| **Sr. No.** | **Statements** | | **Completely agree** | **Agree** | **Disagree** | | **Completely disagree** |
| **605 (i)** | P.A.C.E. training module helped me in better communication at my workplace. | | 1 | 2 | 3 | | 4 |
| **605 (ii)** | I feel more aware about the matters relating to gender bias, stereotype and discrimination at my workplace. | | 1 | 2 | 3 | | 4 |
| **605 (iii)** | I freely discuss on gender and reproductive issues and other problems with my colleagues and seniors. | | 1 | 2 | 3 | | 4 |
| **605 (iv)** | I feel more confident than before in talking about gender discrimination with my colleagues and seniors. | | 1 | 2 | 3 | | 4 |
| **605 (v)** | I discuss with my colleague regarding time and stress management. | | 1 | 2 | 3 | | 4 |
| **605 (vi)** | I try to solve other’s problems. | | 1 | 2 | 3 | | 4 |
| **605 (vii)** | I discuss with my colleague regarding saving options for future needs. | | 1 | 2 | 2 | | 4 |
| **Please tell me about future participation in P.A.C.E. training program** | | | | | | | |
| **606** | If asked again, would you like to participate in such trainings? | | Yes  No  Cannot say | | A  B  C | |  |
| **607** | Would you suggest others to participate in such training programs? | | Yes  No  Cannot say | | A  B  C | |  |
| **608** | Please rate the training session on the scale of 1 to 10.  **Note that 1 being the lowest and 10 being the highest.** | | | | \|  \|  \| \| --- \| --- \| | | |

Thank you
